# The excitement of conducting research and the practical implications

**DOI:** 10.1186/2193-1801-4-S2-K1

**Published:** 2015-11-27

**Authors:** Georges M Halpern

**Affiliations:** Department of Biomedical Sciences, City University of Hong Kong, Hong Kong

Institutions of Higher Education aim at conducting both education and research (medical ones add patients' care). Indeed education without research is simply reciting past obsolete mantras. Research is the discovery of the still unknown, trying to make sense of it, attempting to include it into a current set of projects, discussing it with colleagues (or students!) from other disciplines or background, and ultimately *making our world a better place*. Obviously, since research is neutral, ethics must govern the spirit, the goal, the applications, and what others will eventually use its outcomes for. Not a simple, easy task.

A few people impressed me so strongly that I remain fascinated and devoted to their memory and writings. The two who still guide me - besides my father Bernard, a researcher who introduced the antihistamines, the psycholeptics, immunotherapy of cancer, and more to medicine - are Richard Feynman and Carl Sagan. Richard Feynman famously wrote "Science is the Belief in the Ignorance of the Experts". He meant, and explained that "science - a.k.a. research - is in the making, belongs to the (unknown, yet to be discovered) future, while expertise is based on the past, with in-built obsolescence". This is, in 2015, truer than ever. Technology has changed our/your daily life in ways that your parents could NEVER have imagined. And it's just the beginning: the Elon Musks, the Jack Mas, and all the other game-changers are not going to retire anytime soon, and on the side a new generation of inventors with fangs grooving the soil are waiting.

What we need to do before it's too late is to drastically revise the education model based on reverence, blind Confucian respect of previous generations, religious belief in the textbook scriptures (which were obsolete before being printed). Ethics are essential, but these are based on values that are foreign to law, religion, or even morals. The generation of our students, and future ones, will face a world, challenges and "values" that we today cannot yet imagine, even less conceive. This is not new: Mencius (372-289 BCE) wrote: "One who believes all of a book would be better off without books".

In my own field of medicine (and culinary arts) every single day brings peer-reviewed news that require a 90°, or even 180° change of thinking. Otherwise people suffer -or die. My generation mostly looked passively at this multi-faceted revolution from the side-lines. We were told, "quantum physics is a theory" (*sic*), that wars had been outlawed -and the list goes on. The truth is that *complexity* is more important than ever to apprehend and comprehend, and the thinking based on linear reductionism (from Euclidian to Newtonian) is incapable to explain-even less master-quantum biology, metagenomics or current social interactions. We need the researchers who will make sure that our society develops by mastering these conquests, while remaining harmonious and ethical, embracing everyone. Then we shall be able to rejoice and rest.

